# Efficacy of simvastatin as adjunctive therapy in liver cirrhosis: an updated meta-analysis of randomized controlled trials

**DOI:** 10.1097/MS9.0000000000005302

**Published:** 2026-07-28

**Authors:** Muhammad Fahad Iqbal Khan, Zain Afridi, Muhammad Haris Khan, Muhammad Zahid Anwar, Ali Nawaz, Umama Malik, Muhammad Usama Nawaz, Malik Sheheryar, Muhammad Hamad Ali, Huda Habib, Muhammad Siddique Karimi, Fahad Ali, Shahbaz Ahmad, Fatima Faraz, Sefatullah Sahak

**Affiliations:** a Department of Medicine, Khyber Medical College, Peshawar, Pakistan; b Department of Accident and Emergency, Distric Head Quarters Hospital, Dera Ismail Khan, Pakistan; c Department of Internal Medicine, Lady Reading Hospital, Peshawar, Pakistan; d Department of Medicine, Lady Reading Hospital, Peshawar, Pakistan; e Department of Medicine, Pak International Medical College, Hyatabad, Pakistan; f Department of Medicine, Saidu Medical College, Swat, Pakistan; g Department of Medicine, Lady Reading Hospital, Peshawar, Pakistan; h Department of Medicine, Services Institute of Medical Sciences, Lahore, Pakistan; i Department of Medicine, Saidu Teaching Hospital, Swat, Pakistan; j Department of Medicine, Khyber College of Dentistry, Peshawar, Pakistan; k Department of Medicine, Ayub Medical College, Abbotabad, Pakistan; l Department of Medicine, Rawalpindi Medical University, Rawalpindi, Pakistan; m Faculty of Medicine, Nangarhar University, Jalalabad, Afghanistan

**Keywords:** liver cirrhosis, simvastatin, simvastatin, statins

## Abstract

The clinical benefit of simvastatin as an adjunctive treatment in liver cirrhosis remains inconclusive, with prior syntheses becoming outdated after the publication of the largest trial on the topic. This updated meta-analysis aimed to evaluate the therapeutic impact of simvastatin in cirrhotic patients. A systematic search was conducted in PubMed, Embase, Cochrane Library, and ClinicalTrials.gov using MeSH terms related to “Simvastatin” and “Liver Cirrhosis.” Randomized controlled trials (RCTs) comparing simvastatin to placebo or any other drugs were included. Nine RCTs, including 966 patients, were analyzed. Simvastatin showed no statistically significant effect on all-cause mortality, although a borderline trend toward a 37% relative risk reduction was observed [risk ratio (RR): 0.63; 95% confidence interval (CI): 0.38–1.04; *P* = 0.07], suggesting a potentially clinically meaningful effect. No significant differences were found in bleeding (RR: 0.70; 95% CI: 0.45–1.08), development of ascites (RR: 0.91; 95% CI: 0.69–1.21), hepatorenal syndrome (RR: 0.85; 95% CI: 0.64–1.87), hepatic venous pressure gradient (standardized mean difference [SMD]: −0.19; 95% CI: −0.49 to 0.11), wedged hepatic venous pressure (SMD: −0.11; 95% CI: −0.44 to 0.23), free hepatic venous pressure (SMD: 0.04; 95% CI: −0.21 to 0.29), and adverse events (RR: 1.04; 95% CI: 0.95–1.15), serious adverse events (RR: 0.93; 95% CI: 0.75–1.15), and musculoskeletal adverse events (RR: 1.27; 95% CI: 0.87–1.85). Overall, simvastatin did not demonstrate statistically significant reductions in cirrhosis-related complications. Nevertheless, the observed trend toward lower mortality suggests a possible protective effect that existing trials may be underpowered to confirm. Larger, well-designed RCTs are warranted to clarify its impact on survival outcomes.

HIGHLIGHTS

- Updated meta-analysis of nine randomized controlled trials (RCTs) (966 patients) evaluated simvastatin in liver cirrhosis.
- Simvastatin did not significantly reduce all-cause mortality (risk ratio: 0.63; *P* = 0.07).
- No significant effect on cirrhosis-related complications: bleeding, ascites, or acute kidney injury/hepatorenal syndrome.
- Hepatic hemodynamic parameters (hepatic venous pressure gradient, wedged hepatic venous pressure, free hepatic venous pressure) were unaffected by simvastatin.
- Conclusion: Simvastatin shows no significant clinical benefit in cirrhosis; further large-scale RCTs are needed.

## Introduction

The global burden of liver cirrhosis has significantly increased in recent years^[^1^]^. In 2019 alone, liver cirrhosis accounted for approximately 1.5 million deaths, with a disproportionately higher burden observed among males^[^1,2^]^. The rising incidence is largely attributed to metabolic dysfunction-associated steatotic liver disease (MASLD) and alcohol use^[^3^]^. Cirrhosis involves progressive fibrosis, wherein normal liver parenchyma is replaced by scar tissue, resulting in portal hypertension and hepatic failure. Chronic inflammation and cirrhosis-related complications further aggravate the progression and severity^[^4^]^.

Current treatment strategies primarily aim to manage symptoms rather than reverse the underlying pathology. Standard therapies, including beta-blockers and diuretics, provide symptomatic relief but are insufficient in halting or reversing cirrhotic progression. Similarly, while prophylactic antibiotics reduce the risk of infections, their impact on long-term survival is minimal^[^4^]^. These limitations underscore the need for more effective therapeutic interventions capable of modifying disease trajectory.

Statins have emerged as potential adjunctive agents in chronic liver disease (CLD) and cirrhosis, owing to their pleiotropic effects beyond lipid-lowering. These include anti-inflammatory, antifibrotic, and vasoprotective properties^[^5,6^]^. Statins reduce pro-inflammatory cytokines, inhibit hepatic stellate cell activation – central to fibrosis – and improve endothelial function, thereby lowering portal pressure^[^5,7^]^. Observational studies, especially on simvastatin, have shown associations with improved survival and delayed cirrhosis-related complications^[^8,9^]^.

However, their use in cirrhotic patients remains debated. Concerns over hepatotoxicity have led to cautious use in the past. While newer studies suggest statins are generally safe in compensated cirrhosis, caution is still advised in advanced or decompensated disease^[^10,11^]^. Clinical trial data present a mixed picture: ranging from reductions in mortality and cirrhosis-related complications to no significant long-term benefits^[^8,12^]^. Moreover, findings from trials such as StatLiver indicate that their efficacy may not be universally applicable across all patient cohorts^[^13^]^. This heterogeneity is reflected in the lack of consensus among leading hepatology societies, with some advocating for cautious use in compensated cirrhosis and others emphasizing the need for further research before broader implementation^[^9,11^]^. This inconsistency, along with the absence of unified guidelines, underscores the need for clearer evidence.

Despite emerging interest in statin therapy for cirrhosis, earlier meta-analyses were limited by small sample sizes, the inclusion of non-randomized studies, and outdated data, affecting their relevance to current practice^[^14^]^. Recent randomized controlled trials (RCTs) have strengthened the evidence base, particularly regarding simvastatin’s benefits in compensated cirrhosis. However, the emergence of more recent, large-scale RCTs, most notably Pose *et al*, the largest trial on this topic to date, raises important questions about whether the survival and hemodynamic benefits reported in prior meta-analyses remain accurate, particularly in patients with decompensated cirrhosis, where therapeutic options are most limited^[^15,16^]^. These gaps underscore the need for an updated synthesis of randomized evidence to inform precise, stage-specific therapeutic recommendations. We conducted this systematic review and meta-analysis of RCTs to evaluate the clinical effects of simvastatin in patients with cirrhosis, with a particular focus on mortality, cirrhosis-related complications, and hepatic hemodynamic outcomes.

## Methods

This systematic review and meta-analysis was conducted in accordance with the Preferred Reporting Items for Systematic Reviews and Meta-Analyses (PRISMA) guidelines^[^17^]^. The study protocol is registered on PROSPERO. Artificial intelligence (AI) tools were not utilized at any stage of this review, as reported in accordance with the TITAN Guidelines^[^18^]^.

### Search strategy

A comprehensive literature search was conducted from inception to March 2025 across PubMed/MEDLINE, Embase, and the Cochrane Library. For ongoing or unpublished trials, an additional search was performed on ClinicalTrials.gov. The aim was to identify RCTs evaluating the efficacy of simvastatin, either as monotherapy or in combination with other agents, compared to a control (placebo or other pharmacologic interventions) in patients with liver cirrhosis. The search strategy was developed using the following terms: (“Simvastatin” OR “Zocor” OR “MK-733” OR “MK733” OR “MK 733” OR “Synvinolin”) AND (“Liver Cirrhosis” OR “Hepatic Cirrhosis” OR “Liver Fibrosis” OR “Cirrhosis, Hepatic” OR “Cirrhosis, Liver”). Boolean operators (AND/OR) were employed to optimize the sensitivity and specificity of the search. In addition, reference lists of relevant studies were manually screened (by backward snowballing) to capture any additional eligible trials. No date restrictions were applied.

### Eligibility criteria and study selection

We included studies that met the following criteria: (i) RCTs investigating the therapeutic role of simvastatin (either as a standalone treatment or in combination with other agents) against a control group (receiving placebo or other pharmacologic therapies); (ii) enrolled patients diagnosed with liver cirrhosis; and (iii) reported at least one clinically relevant efficacy or safety outcome. Studies involving animal models or non-randomized or observational methodologies were excluded. The methodological quality of included studies was assessed using the AMSTAR 2 tool, and all items were completed^[^19^]^.

After duplicate removal using Rayyan.ai, two investigators independently screened titles and abstracts according to the predefined eligibility criteria. Full texts of potentially eligible articles were then reviewed in detail to finalize their inclusion in the systematic review and meta-analysis. Discrepancies between reviewers were resolved through discussion or arbitration by a third author. The primary endpoint of this analysis was all-cause mortality. Secondary outcomes included complications such as ascites, bleeding, development of hepatic encephalopathy, hepatorenal syndrome (HRS), hepatic hemodynamic measurements such as free hepatic venous pressure (FHVP), hepatic venous pressure gradient (HVPG), and wedged hepatic venous pressure (WHVP), as well as safety outcomes such as adverse events, serious adverse events, and musculoskeletal adverse events.

### Data extraction

Three reviewers independently extracted data using Excel software. Baseline characteristics and outcome data were extracted. The baseline data included key study details such as the first author, publication year, country of origin, sample sizes for each group, follow-up durations, and baseline clinical parameters. The outcomes data comprised major clinical endpoints such as mortality, complications of ascites, hepatic hemodynamic measurements, and adverse events. All outcomes were assessed at the latest follow-up.

### Risk of bias assessment

The risk of bias in the included RCTs was assessed using the Revised Cochrane Risk of Bias Tool for Randomized Trials (RoB 2), evaluating five domains: the randomization process, deviations from intended interventions, missing outcome data, measurement of the outcome, and selection of reported results^[^20^]^. This assessment was independently conducted by two reviewers and assigned a risk level (low, high, or some concerns). Disagreements between reviewers were resolved through discussion with a third, more experienced author to ensure consensus.

### GRADE assessment

We utilized the GRADE (Grading of Recommendations, Assessment, Development and Evaluation) framework to assess the certainty of evidence across outcomes^[^21^]^. This approach categorizes evidence into four levels, i.e., high, moderate, low, and very low, based on structured evaluation across five domains: risk of bias, inconsistency, indirectness, imprecision, and publication bias. For each outcome, the initial rating for RCTs began at high and was downgraded if concerns were identified in any domain.

### Statistical analysis

Statistical analyses were conducted using Review Manager (version 5.4.1). For dichotomous variables, the Mantel–Haenszel method was applied. Dichotomous outcomes were expressed as risk ratios (RRs) with 95% confidence interval (CI). Continuous data were summarized as standardized mean difference (SMD) values with 95% CI. A random-effects model was used. Summary effects were visualized using forest plots, and between-study heterogeneity was quantified using the *I*^2^ statistic. A funnel plot was constructed for the primary outcome. Publication bias was assessed for outcomes comprising >10 studies using funnel plots, Begg’s, and Egger’s tests. Leave-one-out sensitivity analyses were undertaken. Subgroup analyses were conducted to examine potential sources of heterogeneity, including treatment regimen (simvastatin monotherapy vs. combination therapy), simvastatin dose (predominantly 20 mg vs. predominantly 40 mg), treatment duration (less than 3 months vs. 3 months or more), cirrhosis etiology (predominantly viral vs. predominantly non-viral), and study methodological quality (studies at low risk of bias). Between-subgroup differences were assessed using the interaction p-value, with statistical significance set at *P* < 0.05.

## Results

### Search results

**Figure 1.**
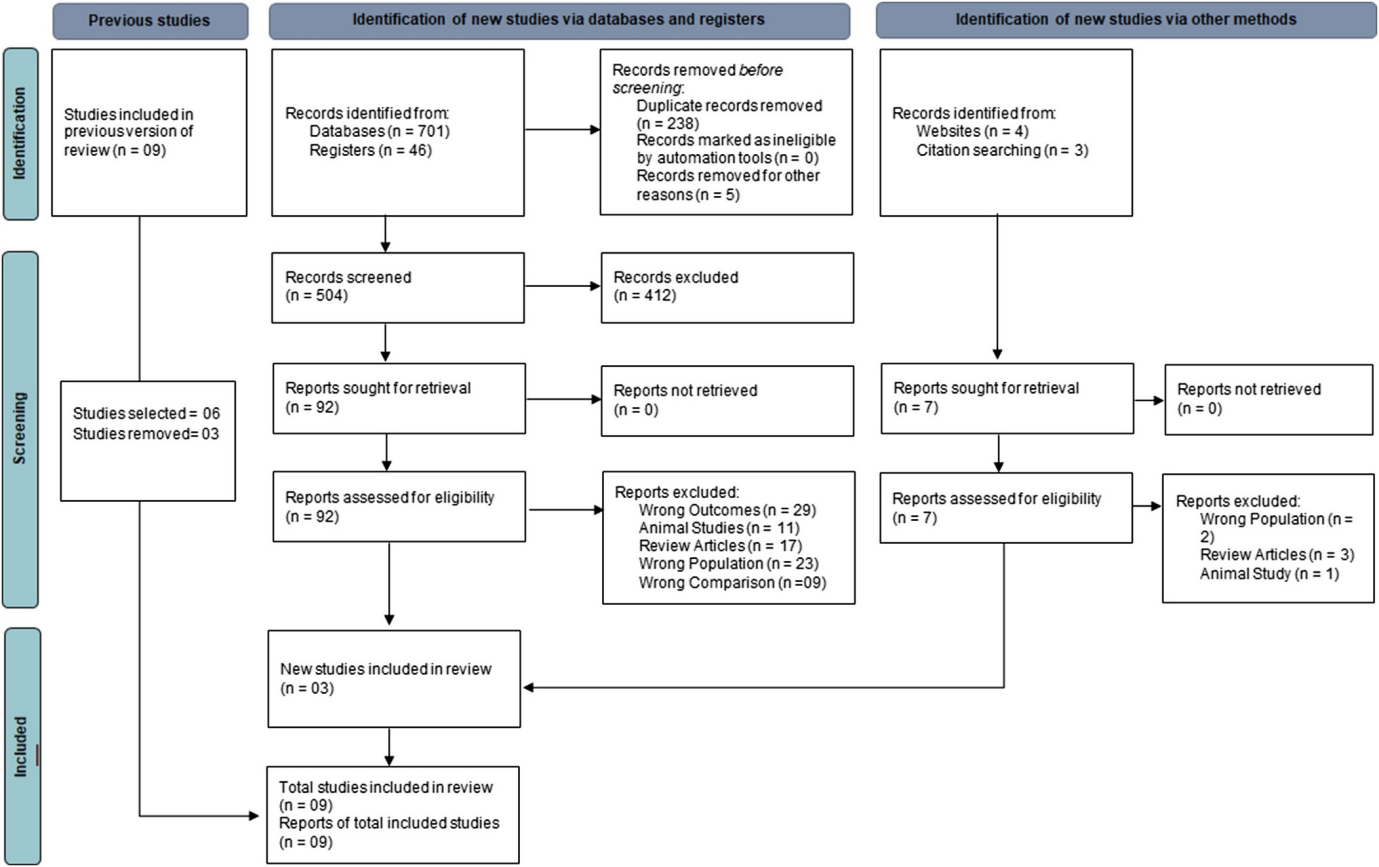
PRISMA Flowchart.

As illustrated in Fig. 1, our comprehensive search of the literature initially identified a total of 754 records. After deduplication and screening for titles and abstracts, we were left with 92 records. Five ongoing clinical trials were also excluded at this stage. Full texts of the remaining articles were evaluated, and a total of 9 RCTs (966 participants) were included^[^15,16,22–28^]^ (Fig. 1). The studies were conducted across more than eight different countries, with four studies originating from Spain^[^16,23,26,27^]^, two from India^[^22,25^]^, one from Europe^[^15^]^, one from Egypt^[^24^]^, and one from Brazil^[^28^]^. Out of these, 473 patients were treated with simvastatin, either as monotherapy or in combination with rifaximin or carvedilol, while the remaining 493 patients received comparators, which included carvedilol alone, placebo, or both.

### Study characteristics

A total of nine RCTs were included, with broadly comparable baseline characteristics between the two groups. Sample sizes, age, and sex distribution were generally similar. Cirrhosis was attributed to various etiologies, including alcohol, hepatitis B, hepatitis C, primary biliary cirrhosis, MASLD, among others. Detailed baseline characteristics are presented in Table 1.

**Table 1:** Characteristics of studies and patients in meta-analysis.

Study ID	Country/Area	Intervention drug (dose, ROA, frequency, duration)	Participants, *n* (I/C)	Male, % (I/C)	Age (yrs)	Child–Pugh A/B/C (%)	Child–Pugh score	MELD score	Etiology	Albumin (mg/dL)	INR	Follow-up time for outcomes
		Control drug (dose, ROA, frequency, duration										
Pose 2025^[^15^]^	Europe	Simvastatin (20 mg, oral, once per day, 12 months) and Rifaximin (400 mg, oral, 3 times per day, 12 months)	237 (117/120)	75.2/68.3	58/56	(0/82.9/17.0)/(0/80.8/19.2)	(97/97)/(20/ 23)	14/14	Alcohol (97/92), MASLD (15/24), HCV 13/6), HBV (6/8), Cryptogenic (5/3), Other (7/17)	3400/3400	1.3/1.4	12 Months
		Placebo (matched appearance, oral, daily, for 12 months)										
Abraldes 2016^[^23^]^	Spain	Simvastatin (20 mg for 15 days then 40 mg, oral, once per day, 24 months)	147 (69/78)	65.2/67.9	57.42/57.62	(15/68/17)/(24/62/14)	7.93/7.68	10.15/10.03	Alcohol (49/55), HCV (19/17), HBV (1/2), Primary Biliary Cirrhosis (3/0),NASH (1/4), Other (5/7)	2820/2940	1.41/1.42	24 Months
		Placebo (20 mg for 15 days then 40 mg, oral, once per day, 24 months)										
Abraldes 2009^[^27^]^	Spain	Simvastatin (20 mg for 15 days then 40 mg, Oral, -, 30 days)	55 (28/27)	60.1/77.8	58 ± 10/56 ± 10	(64.3/35.7/0)	(59.3/29.6/11.1)	N/A	Alcohol (11/12), HCV (14/13), HBV (0/2), Others (3/0)	3800 ± 500/3900 ± 700	N/A	1 Month
		Placebo (NA, oral, NA, 30 days)										
Elwan 2018^[^24^]^	Egypt	Simvastatin (20 mg for 2 weeks then 40 mg for 2 weeks, oral, once per day, 4 weeks)	40 (20/20)	50/80	51.5 ± 6.7/50.8 ± 7.0	(15/60/25)/(5/45/50)	N/A	N/A	HCV	2780 ± 509 /2710 ± 434	N/A	4 Weeks
		-										
Pollo-Flores 2015^[^28^]^	Brazil	Simvastatin (40 mg, oral, once per day, 3 months)	34 (14/20)	57/50	56.5/58.5	(8/5/1)/(14/5/1)	6;2.7/ 6;3	10/10.5	HCV (58%), Alcoholic Liver Disease (17%), HBV (17%), Autoimmune Hepatitis (8%)	3400/3500	1.3/1.3	6 Months
		Placebo (NA, oral, once per day, 3 months)										
Vijayaraghavan 2020^[^25^]^	India	Simvastatin (40 mg, oral, once per day, 3 months) and Carvedilol (12.5 mg, oral, twice per day, 3 months)	220 (110/110)	87.2/78.1	51.1/52.5	(33.6/50.9/15.5)/(43.6/38.2/18.2)	N/A	14.4/13.9	Alcohol (44/39), NASH (48/42), HBV (7/10), HCV (6/13)	3290/3270	1.58/1.56	3 Months
		Carvedilol (12.5 mg, oral, twice per day, 3 months)										
Jha 2019^[^22^]^	India	Simvastatin (40 mg, oral, NA, 50 weeks) and Carvedilol (10 mg, oral, NA, 50 weeks)	134 (65/69)	69.2/73.9	44.86 ± 15.40/46.02 ± 14.18	(9.2/70.8/20)/(13/62.4/24.6)	8.29 ± 1.29/8.52 ± 1.54	15.03 ± 6.46/16.49 ± 5.75	Alcohol (17/25), HBV (19/23), HCV (6/3), Cryptogenic (18/14), Other (5/4)	2780 ± 490/2660 ± 410	1.44 ± 0.44/1.52 ± 0.38	49.05 ± 25.74 Weeks
		Carvedilol (10.01 mg, oral, NA, 48.21)										
Zafra 2004^[^26^]^	Spain	Simvastatin (40 mg, oral, twice only: 12 and 1 h before test meal, 1-day protocol)	17 (9/8)	88.8/75	65 ± 7/59 ± 12	N/A	7.7 ± 2.1/6.8 ± 1.8	N/A	N/A	3500 ± 500/3300 ± 700	N/A	45 minutes
		Lactase (10 mg, oral, twice: 12 and 1 h before test meal, 1-day protocol)										
Tapias 2024	Spain	Carvedilol (17.9 mg, oral, NA, 6–8 weeks) and Simvastatin (20 mg for 2 weeks and then 40 mg, oral, NA, 4–6 weeks)	82 (41/41)	64/71	62 ± 10/63 ± 11.3	22/-/-	6.2 ± 1.28/6.5 ± 1.5	9.7 ± 2.7/10.3 ± 3.4	Alcohol (16/18), HCV (14/14), Alcohol + HCV (2/5), MASLD (5/2), Others (4/2)	3380 ± 500/3390 ± 600	1.19 ± 0.2/1.24 ± 0.2	6 to 8 Weeks
		Carvedilol (17.7 mg, oral, NA, 6–8 weeks) and Placebo (20 mg for 2 weeks and then 40 mg, oral, NA, 4–6 weeks)										

### Bias assessment

**Figure 2.**
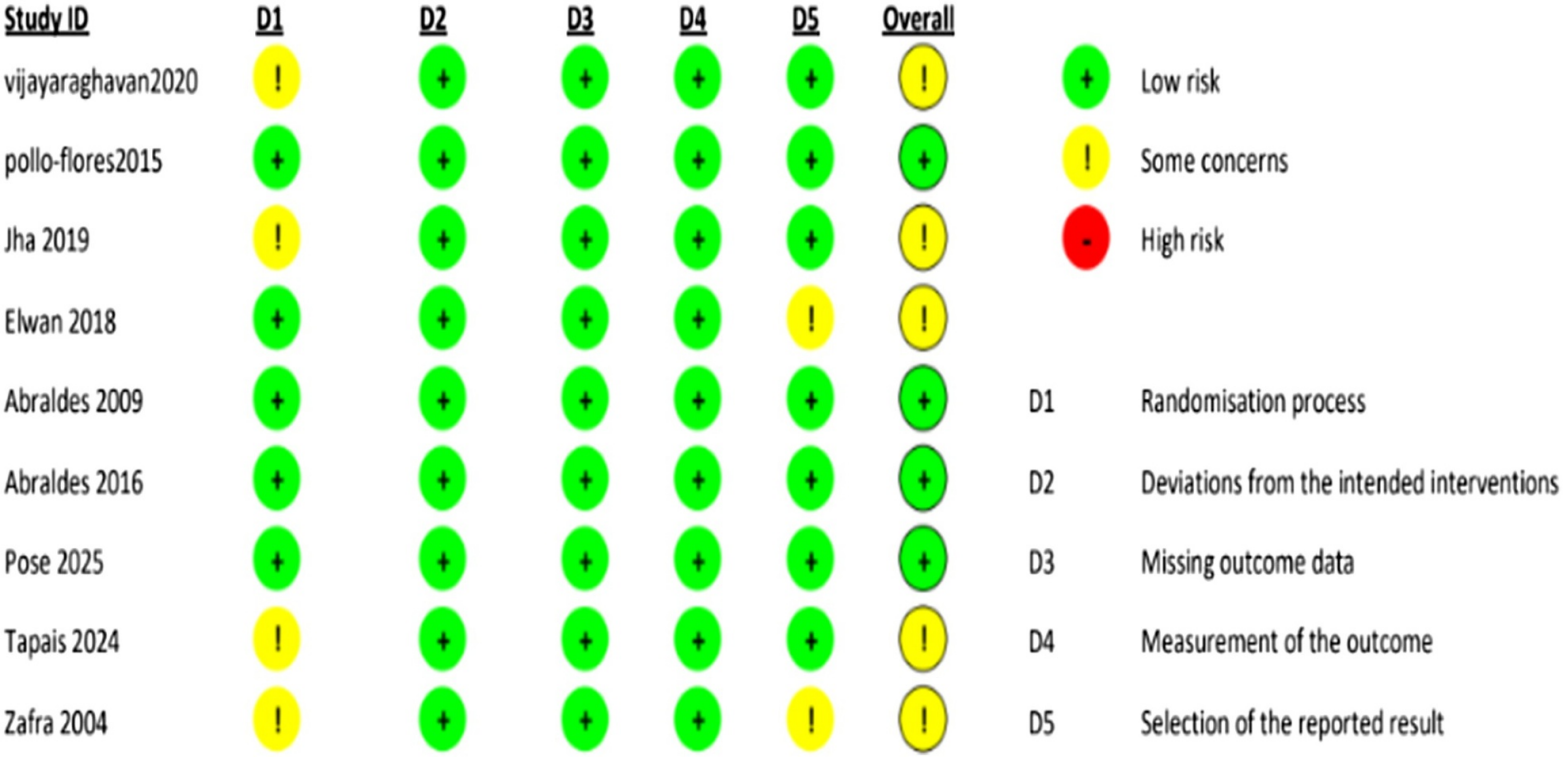
Risk of bias assessment.

The risk of bias in the included studies was assessed using Cochrane ROB 2.0^[^17^]^. Out of the nine RCTs, five exhibited some concerns, particularly in the randomization process and the selection of the reported result. The remaining four studies showed a low risk of bias. The risk of bias assessment of the included studies is depicted in Fig. 2.

### Certainty of evidence

In our analysis, all of the aforementioned outcomes were graded as having moderate certainty of evidence. The primary reason for downgrading was serious imprecision. All judgments and certainty levels are summarized in Supplemental Digital Content Table 1, available at: https://links.lww.com/MS9/B317.

## Meta-analysis of primary and secondary outcomes

### Mortality

**Figure 3.**
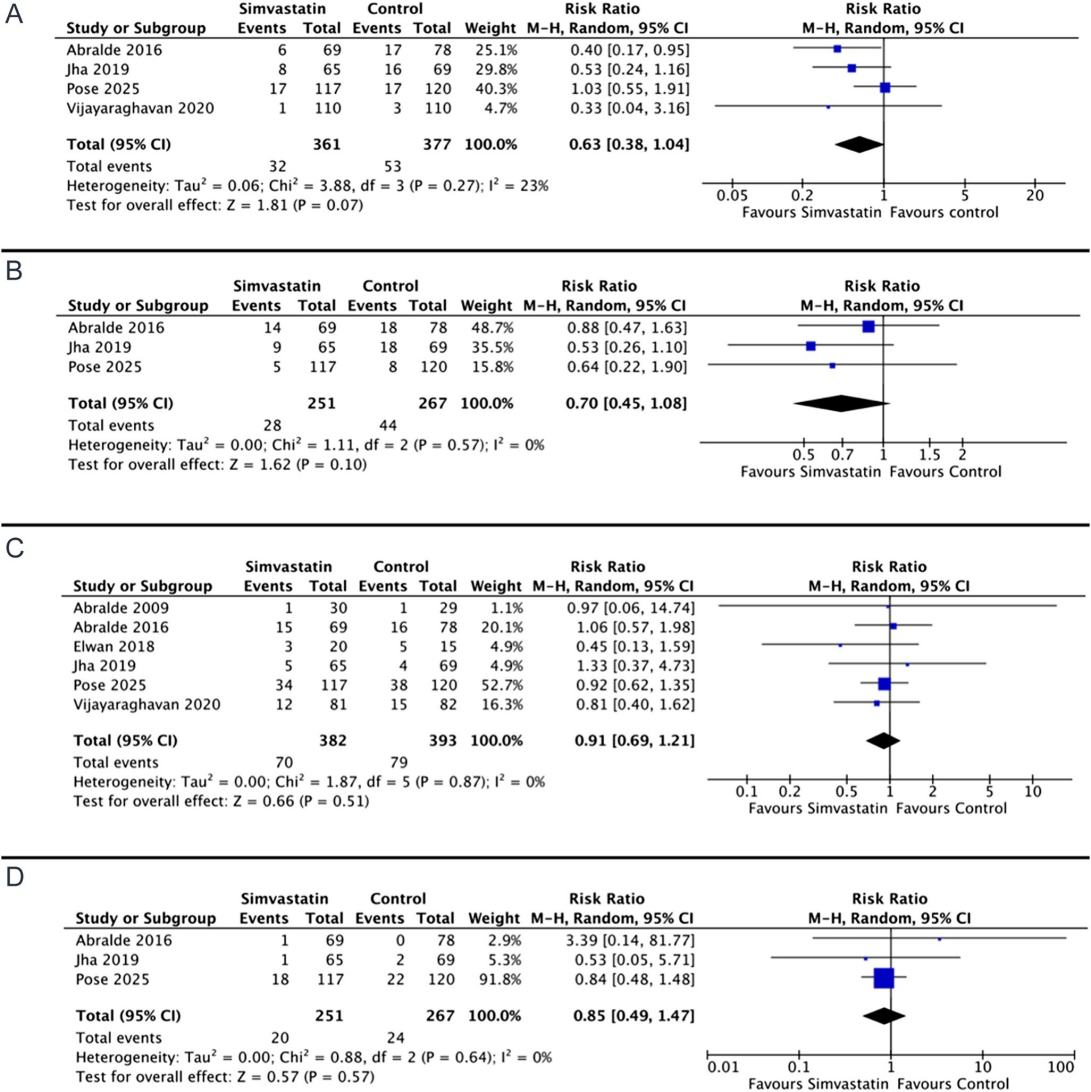
Forest plots demonstrating the comparison of Simvastatin to control groups: (A) Mortality; (B) Bleeding; (C) Ascites; (D) Hepatorenal Syndrome.

This outcome was assessed in four studies with a combined sample size of 738 participants (361 in the simvastatin group and 377 in the placebo group). The pooled analysis indicated no statistically significant reduction in mortality in the simvastatin group compared to placebo (RR = 0.63; 95% CI: 0.38–1.04; *P* = 0.07). Heterogeneity among studies was low and not statistically significant (*I*^2^ = 23%), indicating a high level of consistency across studies (Fig. 3a). When Pose *et al* was excluded in the sensitivity analysis, the result became significant (RR = 0.46; 95% CI: 0.26–0.80; *P* = 0.006; *I*^2^ = 0%). All subgroup analyses revealed no significant interactions across all examined subgroups, suggesting the effect was consistent regardless of subgroup characteristics (Supplementary Digital Content Table 2, available at: https://links.lww.com/MS9/B305).

### Bleeding

Three studies assessed bleeding outcomes in a total of 518 participants. The analysis demonstrated a non-significant reduction in bleeding in the simvastatin group compared to placebo (RR = 0.70; 95% CI: 0.45–1.08; *P* = 0.10). No heterogeneity was observed (*I*^2^ = 0%) (Fig. 3b). Results of the sensitivity analysis were comparable to the primary analysis. All subgroup analyses revealed no significant interactions across all examined subgroups, suggesting the effect was consistent regardless of subgroup characteristics (Supplementary Digital Content Table 2, available at: https://links.lww.com/MS9/B305).

### Ascites

Ascites was evaluated in six studies, including 775 participants. No statistically significant difference was observed between the two groups (RR = 0.91; 95% CI: 0.69–1.21; *P* = 0.51). The analysis showed excellent homogeneity (*I*^2^ = 0%) (Fig. 3c). Results of the sensitivity analysis were comparable to the primary analysis. All subgroup analyses revealed no significant interactions across all examined subgroups, suggesting the effect was consistent regardless of subgroup characteristics (Supplementary Digital Content Table 2, available at: https://links.lww.com/MS9/B305).

### Hepatorenal syndrome

Three studies reported the incidence of HRS. The risk did not differ significantly between groups (RR = 0.85; 95% CI: 0.49–1.47; *P* = 0.57), with no between-study variation (*I*^2^ = 0%) (Fig. 3d). Results of the sensitivity analysis were comparable to the primary analysis. All subgroup analyses revealed no significant interactions across all examined subgroups, suggesting the effect was consistent regardless of subgroup characteristics (Supplementary Digital Content Table 2, available at: https://links.lww.com/MS9/B305).

#### Hepatic venous pressure gradient

**Figure 4.**
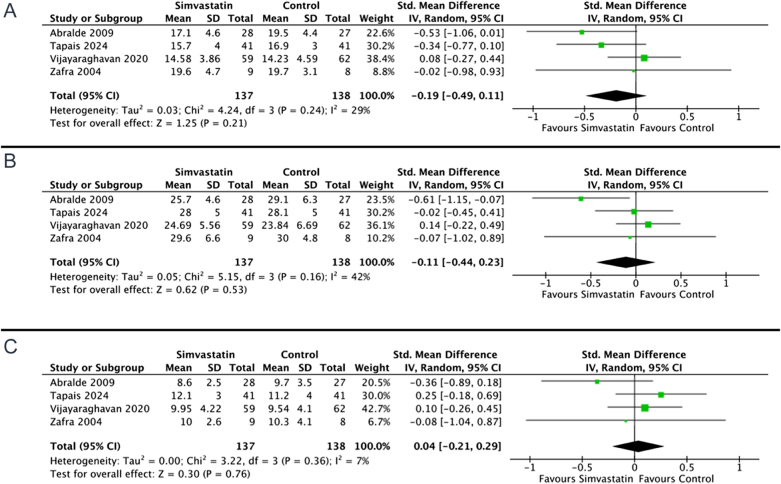
Forest plots demonstrating a comparison of simvastatin to control groups in hepatic hemodynamic measurements: (A) Hepatic Venous Portal Gradient (HVPG); (B) wedged hepatic venous pressure (WHVP); (C) free hepatic venous pressure (FHVP).

HVPG was assessed in four studies with a total sample size of 275 participants. The pooled analysis revealed no significant difference in HVPG between the two groups [standardized mean difference (SMD) = −0.19; 95% CI: −0.49 to 0.11; *P* = 0.21). Heterogeneity was low (*I*^2^ = 29%) (Fig. 4a). Results of the sensitivity analysis were comparable to the primary analysis. All subgroup analyses revealed no significant interactions across all examined subgroups, suggesting the effect was consistent regardless of subgroup characteristics (Supplementary Digital Content Table 2, available at: https://links.lww.com/MS9/B305).

#### Wedged hepatic venous pressure

WHVP was analyzed in four studies involving 275 participants. The findings showed lower WHVP in the simvastatin group (SMD = −0.11; 95% CI: −0.44 to 0.23; *P* = 0.53); however, the difference was not statistically significant. Moderate heterogeneity was observed (*I*^2^ = 42%) (Fig. 4b). When Abralde *et al* (2009) was excluded in the sensitivity analysis, heterogeneity became negligible (SMD = −0.11; 95% CI: −0.44 to 0.23; *P* = 0.64; *I*^2^ = 0%). All subgroup analyses revealed no significant interactions across all examined subgroups, suggesting the effect was consistent regardless of subgroup characteristics (Supplementary Digital Content Table 2, available at: https://links.lww.com/MS9/B305).

#### Free wedged hepatic venous pressure

FWHVP outcomes were reported in four studies. The analysis showed no significant difference between the two groups (SMD = 0.04; 95% CI: −0.21 to 0.29; *P* = 0.53). Heterogeneity was minimal (*I*^2^ = 7%) (Fig. 4c). Results of the sensitivity analysis were comparable to the primary analysis. All subgroup analyses revealed no significant interactions across all examined subgroups, suggesting the effect was consistent regardless of subgroup characteristics (Supplementary Digital Content Table 2, available at: https://links.lww.com/MS9/B305).

### Adverse events

**Figure 5.**
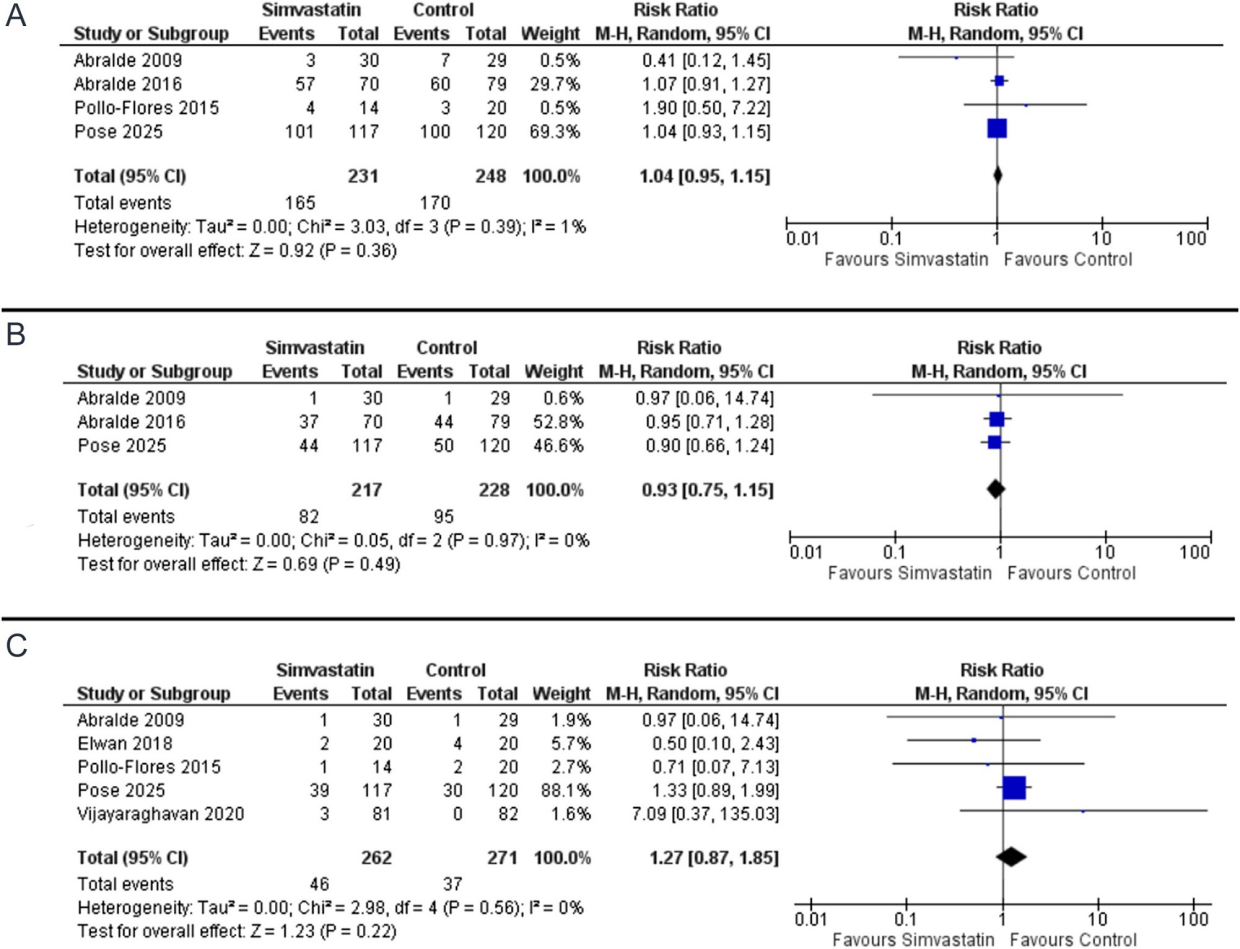
Forest plots demonstrating the comparison of Simvastatin to control groups in safety outcomes: (A) adverse events; (B) serious adverse events; (C) musculoskeletal adverse events.

Adverse events were reported in four studies. The analysis showed no significant difference between the two groups (RR = 1.04; 95% CI: 0.95–1.15; *P* = 0.36). Heterogeneity was minimal (*I*^2^ = 1%) (Fig. 5a). Results of the sensitivity analysis were comparable to the primary analysis. All subgroup analyses revealed no significant interactions across all examined subgroups, suggesting the effect was consistent regardless of subgroup characteristics (Supplementary Digital Content Table 2, available at: https://links.lww.com/MS9/B305).

### Serious adverse events

Three studies reported serious adverse events. The risk did not differ significantly between groups (RR = 0.93; 95% CI: 0.75–1.15; *P* = 0.49), with no between-study variation (*I*^2^ = 0%) (Fig. 5b). Results of the sensitivity analysis were comparable to the primary analysis. All subgroup analyses revealed no significant interactions across all examined subgroups, suggesting the effect was consistent regardless of subgroup characteristics (Supplementary Digital Content Table 2, available at: https://links.lww.com/MS9/B305).

### Musculoskeletal adverse events

Three studies reported musculoskeletal adverse events. The risk did not differ significantly between groups (RR = 1.27; 95% CI: 0.87–1.85; *P* = 0.22), with no between-study variation (*I*^2^ = 0%) (Fig. 5c). Results of the sensitivity analysis were comparable to the primary analysis. All subgroup analyses revealed no significant interactions across all examined subgroups, suggesting the effect was consistent regardless of subgroup characteristics (Supplementary Digital Content Table 2, available at: https://links.lww.com/MS9/B305).

## Discussion

Liver cirrhosis remains a leading cause of global morbidity and mortality, particularly when decompensation occurs, leading to complications like variceal bleeding, ascites, and renal dysfunction^[^29,30^]^. Simvastatin, beyond its lipid-lowering properties, exerts pleiotropic effects that may be particularly beneficial in CLD, chronic obstructive pulmonary disease, kidney disease, pancreatitis, or erectile dysfunction^[^9^]^. The underlying proposed mechanism is the modulation of endothelial nitric oxide synthase, inhibition of Rho-kinase pathways, and improved hepatic microcirculation^[^31–33^]^.

Our pooled analysis showed a non-significant reduction in all-cause mortality with simvastatin. No significant effects were observed in cirrhosis complications, hemodynamic parameters, or adverse events. These findings update and refine those of the previous meta-analysis by Zhang *et al*, which had reported a significant reduction in mortality and bleeding risk based on nine trials with 648 participants^[^34^]^.

While the prior meta-analysis by Zhang *et al* suggested that simvastatin significantly reduced mortality (RR: 0.46; 95% CI: 0.29–0.73; *P* < 0.01), our updated results temper this conclusion^[^34^]^. Our pooled estimate of RR 0.63 (95% CI: 0.38–1.04; *P* = 0.07) reflects both a shift in the point estimate toward the null and a widening of the CI, resulting in a borderline but non-significant reduction. This attenuation is driven by the exclusion of non-randomized studies in our meta-analysis, as well as the inclusion of the trial by Pose *et al*, the largest RCT on this topic to date, which carried considerable statistical weight and shifted the pooled estimate toward the null^[^15^]^. Nevertheless, a 37% relative risk reduction, despite being statistically insignificant, raises the possibility that the current body of evidence remains insufficiently powered to detect a true mortality benefit, and a type II error cannot be excluded given the limited cumulative sample size and low event rates across included trials. Furthermore, Pose *et al* employed a lower dose of simvastatin (20 mg) compared to the more standard 40 mg dose, which may account for the difference. This finding is especially relevant in the context of decompensated cirrhosis, with limited therapeutic options and poor clinical outcomes. The proposed mechanisms underlying these benefits include enhanced endothelial nitric oxide production, reduction in intrahepatic vascular resistance, and antifibrotic activity, all contributing to improved hepatic perfusion and delayed progression of liver dysfunction^[^32^]^. However, our pooled synthesis reports no improvement in survival based on the currently available randomized data, irrespective of treatment duration or cirrhosis etiology.

Simvastatin’s impact on clinical complications, such as ascites and HRS, also appeared negligible. Prior analyses had hypothesized indirect benefits via improved renal perfusion or vascular resistance modulation, but the absence of significant effects in our updated synthesis suggests these pathways may not be sufficiently robust in advanced disease settings^[^16,28,35^]^. Similarly, although previous animal models and small trials hinted at reduced bleeding risk with statins, our findings did not confirm a statistically significant reduction in bleeding events^[^16,36^]^. Moreover, in contrast to initial concerns regarding potential immunosuppressive effects of statins, the data do not support an increased risk of infections, such as spontaneous bacterial peritonitis^[^35^]^. These findings lend support to the safety of simvastatin in a population inherently vulnerable to both infectious and hemorrhagic complications. Our study revealed a non-significant reduction in the risk of HRS, defined as a rise in serum creatinine of 50% or more from baseline, reaching a final value greater than 1.5 mg/dL (133 μmol/L)^[^37^]^. Studies have proposed renal protection due to statins’ anti-inflammatory and endothelial-stabilizing effects, but clinical confirmation is very limited^[^9^]^.

Our findings on HVPG reduction contrast with earlier reports. While the previous analysis by Zhang *et al* and Wan *et al* noted a trend toward HVPG reduction, our updated meta-analysis found no significant difference in HVPG, nor in FHVP or WHVP^[^34,38^]^. These differences may reflect the limited number of trials reporting hemodynamic outcomes, heterogeneity in patient populations, inadequate dosages and durations, and concurrent use of other portal pressure-lowering agents. Theoretically, simvastatin-induced increases in hepatic nitric oxide products and improved response to beta-blockers should improve hemodynamic outcomes. These mechanisms have been shown to reduce portal pressure and inflammation in preclinical models and are also suggested in human studies^[^26^]^. Nevertheless, the currently available randomized data do not reflect this.

Importantly, safety remains a key consideration. Both our meta-analysis and the previous meta-analysis by Zhang *et al* conclude that simvastatin was generally safe in cirrhotic patients, citing a low incidence of adverse effects^[^34^]^. However, our analysis did not include a formal synthesis of hepatotoxicity due to inconsistency in reporting and definitions employed. This gap carries particular clinical weight in the context of cirrhosis, where baseline hepatic dysfunction renders patients inherently more susceptible to drug-induced liver injury. The heterogeneity in how included trials monitored and defined liver-related adverse events means that a reassuring safety profile cannot be reliably concluded from the available data alone. Although our pooled analysis did not report a significant increase in musculoskeletal adverse events, rare events such as rhabdomyolysis (as reported by Abraldes *et al*) and elevated liver enzymes in vulnerable subgroups (e.g., Child–Pugh C) warrant cautious use and close biochemical monitoring^[^23,27^]^.

Although our meta-analysis did not report significant results with simvastatin, further research may unveil the impact of simvastatin in patient populations that would theoretically derive more benefit from this treatment. Patients with decompensated liver cirrhosis with preserved hepatic reserve may be one potential subgroup that could benefit from simvastatin^[^39^]^. Similarly, longer treatment durations and the potential additive effect of simvastatin in patients receiving beta-blocker therapy could potentially be explored through future research^[^40^]^.

CLDs pose complex diagnostic, prognostic, and therapeutic challenges that require integration of multimodal data, including laboratory values, imaging, histopathology, and longitudinal clinical trends. Current care pathways frequently rely on sequential specialist consultations, which can fragment decision-making and introduce delays in timely risk stratification and treatment optimization. AI agents, defined as autonomous systems capable of reasoning, learning, and collaborative decision-making, offer a transformative alternative through distributed, network-based problem-solving. AI has already demonstrated substantial progress in similarly complex patient populations where large volumes of heterogeneous data must be synthesized for accurate prediction and clinical guidance^[^41,42^]^. Multiple AI-driven agent platforms are now under development to support prognostication, assess the risk of malignant transformation such as hepatocellular carcinoma, and enhance clinical decision support, with the potential to fundamentally reshape CLD management^[^43^]^.

Our review has several strengths. First, the trials included in this meta-analysis were geographically diverse, with studies conducted in the UK, Spain, Egypt, Brazil, and India. Notably, two of the larger trials originated from India, ensuring representation from South Asian populations. Second, the analyses revealed little to no heterogeneity, indicating consistent results across the studies.

This meta-analysis also has notable limitations. First, limited statistical power due to small sample sizes and low event rates raises the possibility of a type II error, particularly for mortality. Second, the included studies varied in treatment characteristics, such as concomitant medication, intervention and control group dose, and duration. Although subgroup analyses were undertaken to address these sources of heterogeneity and revealed consistent results, a low number of studies in each subgroup substantially limited the statistical power of the interaction tests and the reliability of the findings. Furthermore, even within subgroups, considerable clinical variability remained, as studies could not be fully disaggregated by all relevant treatment characteristics simultaneously. As such, the subgroup analyses’ results should be interpreted with caution. Third, inconsistent documentation of outcomes and variations in follow-up duration may introduce heterogeneity. The lack of standardized definitions for hepatotoxicity is a major limitation, particularly for cirrhosis, where margins for additional hepatic insult are narrow. Fourth, several patient characteristics could potentially confound our results. The impact of the Child–Pugh score and comorbidities on statin efficacy could not be addressed. The included studies also inconsistently reported concomitant medications, particularly beta-blockers. This precluded any adjustment for background therapy in our analyses and represents a meaningful limitation, as patients already receiving beta-blockers may exhibit a ceiling effect whereby the additional hemodynamic benefit of simvastatin is attenuated, potentially contributing to the non-significant findings observed. Fifth, the overall certainty of evidence was rated as moderate according to the GRADE assessment for all outcomes, downgraded primarily due to imprecision stemming from limited sample sizes, which resulted in wide CIs and reduced the precision of pooled estimates. Methodological limitations were also identified across several included studies upon risk of bias assessment, particularly concerning inadequate or unclear randomization procedures, which raises concerns about potential selection bias, and incomplete or selective outcome reporting, which may have led to an overestimation or underestimation of the true treatment effect. Collectively, these methodological shortcomings reduce confidence in the robustness of the findings and highlight the need for larger, methodologically rigorous RCTs to generate higher-certainty evidence. Sixth, publication bias could not be assessed due to the inclusion of fewer than 10 studies per outcome and therefore cannot be excluded as a potential source of bias. Seventh, the pooling of studies evaluating acute and chronic simvastatin effects on HVPG (one and three studies, respectively), which may reflect distinct hemodynamic mechanisms, potentially dilutes the true treatment effect.

## Conclusion

In summary, this updated meta-analysis does not confirm a statistically significant mortality benefit of simvastatin in cirrhosis, although potentially underpowered results suggest a possible protective trend remains biologically and clinically plausible. The drug also did not significantly impact complications of liver cirrhosis, hemodynamic parameters, or adverse events. These findings call for a more nuanced interpretation of earlier results and suggest the need for further large-scale RCTs to better define the subgroups most likely to benefit, optimal dosing strategies, and long-term safety, with standardized hepatotoxicity reporting. Based on the currently available evidence, simvastatin cannot be recommended in liver cirrhosis.

## Data Availability

The datasets will be provided by the corresponding author upon reasonable request.

## Research registration unique identification number (UIN)

Prospero: CRD420251080119.
